# The Power of *WNT5A* and *FZD3* Gene Expression and Methylation Status in the Diagnosis–Treatment–Cause Triangle in Tension-Type Headache

**DOI:** 10.3390/cimb46110758

**Published:** 2024-11-10

**Authors:** Ferhat Kılıçaslan, Sırma Geyik, Şenay Görücü Yılmaz

**Affiliations:** 1 Department of Neurology, Gaziantep University, Gaziantep 27310, Turkey; ferhatkilicaslan93@gmail.com (F.K.); sirmageyik@gantep.edu.tr (S.G.); 2 Department of Nutrition and Dietetics, Gaziantep University, Gaziantep 27310, Turkey

**Keywords:** tension-type headache, WNT5A, FZD3, gene expression, methylation, ROC curve

## Abstract

DNA methylation is the epigenetic pathway controlling cellular gene expression. Methylation is a natural and cellular epigenetic mechanism for gene silencing. The fact that the genes that the cell decides to be silent do not speak or begin to speak may coincide with diseases. For explanatory evidence, changes at the DNA level can provide realistic information. Wnt/β-catenin signaling has an important role in the pain process. For this purpose, we investigated the relationship between clinical data, wingless-type MMTV integration site family, member 5A (*WNT5A*), and Frizzled Class Receptor 3 (*FZD3*) gene methylation and expression in a cohort of tension-type headache (TTH) patients (N = 130) and healthy control (N = 117) individuals. Comorbidities were evaluated. Methylation profiling was performed using Real-Time PCR with a TaqMan primer-probe. The diagnostic power (receiver operating characteristic—ROC) was determined according to the expression and methylation status. Ultimately, *WNT5A* was found to be upregulated and hypermethylated, and *FZD3* was found to be upregulated and hypomethylated. Finally, the area under the curve (AUC) data for *FZD3* upregulation (0.983) and hypomethylation (0.866) showed diagnostic values. *WNT5A* and *FZD3* may contribute to the pathogenesis of the disease depending on their expression and methylation profile during the TTH process. At the same time, diagnostic powers have the potential to be a resource for early treatment and new therapeutic approaches.

## 1. Introduction

Methylation is an essential epigenetic regulation that plays an important role in gene dosage from the fetal period and in the regulation of the biological program. Methylation activity from X chromosome inactivation to the timing of gene expression contributes to genome stability during cell differentiation and fetal development. The most important evidence showing the gene regulation ability of the methylation mechanism comes from studies showing that methyl sites near the promoter region vary according to cell type. Promoter methylation status is associated with decreased, increased, or absent transcription [1]. In addition to methylation differences between individuals, methylation patterns also differ in an individual’s tissues. The difference in general and specific methylation levels is observed between cancer cells and healthy cells. This fact suggests the association of methylation errors with various diseases, considering the important role of methylation states in gene expression.

The puzzle researchers need to solve is quite large, considering the entire genome. Data obtained from studies examining diseases such as cancer, muscular dystrophy, lupus, and congenital defects thought to occur as a result of stigma errors, have helped understand diseases with more complex mechanisms [2]. Although the pathogenesis is not clear in the best-defined TTH, the effect of abnormal DNA methylations is known. The benefit of folate, which is effective in DNA methylation, in migraine has been demonstrated by changes in the methylenetetrahydrofolate reductase gene. Therefore, a folate-containing diet that targets DNA methylation may indirectly target migraine [3]. Although there is no epigenetic-based preventive treatment for migraine, studies are continuing persistently. One study investigated DNA methylation changes in the episodic headache–chronic headache transition. In the 485,000 CpG sites scanned, two genes associated with disease and methylation were identified (*SH2D5* and *NPTX2*). Additionally, pathway analyses demonstrated the influence of Ca binding and estrogen processes [4].

Genome-wide methylation profiling studies identified 62 differential methyl regions that may be responsible for migraine [5]. These studies indicated that there may be a relationship between pain and methylation, but specific genes and methylation profiles are needed for strong evidence. When we narrow the pain down to cluster type, we can find the opportunity to work in a more specific way. Cluster headaches have a characteristic cycle that recurs on a 24-hour cycle or seasonally at certain times of the year. The hypothalamus, which is essential for the functioning of the circadian clock, may be effective in the perception of pain. Regional anomalies may help explain the cyclic movement and timing of this pain. A common process or focus can be identified based on triggers of cluster headaches, such as vasodilators, histamine, and tobacco exposure [6].

The methylation status in the promoter region of the *CALCA* gene is low in episodic migraine. This condition may be associated with various clinical features [7]. The basic mechanisms responsible for this pain include circadian rhythm, genetic factors, histamine, mast cell increase, and trigeminal nerve stimulation. WNT5A is involved in the regulation of the nervous system and is critical in many types of neuropathic pain [8]. The methylation status of *WNT5A* in TTH and its relationship with the expression change of the gene may indicate physiological processes and its potential as a treatment target. FZD3 family members are receptors of WNT (https://www.omim.org/entry/606143 (accessed on 20 November 2023)). Wnt-Fzd signaling causes sensitization of peripheral sensory neurons [9]. Data on the functions and pathology of the FZD are limited. FZD3, the WNT ligand, is a molecule that should be studied to evaluate the function of the mechanisms it mediates. In addition to the role that the WNT pathway plays in many types of neuropathic pain and pain originating from the nervous system, its role in the trigeminovascular system associated with cluster headaches suggests that it may be related to the disease [10]. Evaluating the activities of *FZD3* and its ligand *WNT5A* will help to understand the functioning of these genes and their clinical relationship in TTH.

Additionally, the power of epigenetic mechanisms in gene regulation can be shown to be linked to methylation. Thus, it can be presented as a potential therapeutic target and the diagnostic value of expressional methylation statuses can be demonstrated in cohorts. We evaluated the *WNT5A* and *FZD3* gene expression, methylation status, relationship with clinical data, and diagnostic power in whole blood samples of TTH patients.

## 2. Materials and Methods

### 2.1. Ethical Standards

This study was approved by the Gaziantep University Clinical Research Ethics Committee (protocol number: 2021/360; date, 26 January 2022). All participants signed the voluntary consent form. The study was conducted following the Declaration of Helsinki.

### 2.2. Study Population

The study group consisted of patients who applied to the Gaziantep University Neurology clinic and healthy volunteers who were not diagnosed with migraine according to the International Classification of Headache Disorders 3rd beta version (ICHD-3beta) [11]. A total of 247 individuals (117 healthy controls and 130 TTH patients) was included in the study. *Inclusion criteria*: The diagnosis was given according to (ICHD)-3 criteria [12]. The study population consisted of male and female individuals between the ages of 17 and 82. Volunteers who were determined not to have secondary headaches by checking complete blood count, blood biochemistry, thyroid function tests, vitamin B12, and folic acid blood levels, and who did not have any other causes of headache such as subdural hematoma or intracranial mass in the neuroradiological examination performed with cranial CT and MRI, and individuals who were not diagnosed with tension-type or other primary headaches were included in the study. *Exclusion criteria*: Individuals who were not diagnosed with tension-type headache, who had other reasons that could explain the pain in laboratory examinations or imaging techniques, and pregnant and breastfeeding individuals were not included in the study. *Healthy control* patients with other primary or secondary headaches and migraine history were not included in the healthy group. *Epidemiological data*: In the patient group, age, gender, weight, height, Hb, WBC, platelet, urea, Cre, AST, ALT, B12, folate, TSH, number of days with pain, attack severity, pain location, smoking, alcohol, chronic diseases, and analgesic use were evaluated. Glucose and HbA1c were not evaluated because only two patients had a diagnosis of diabetes.

### 2.3. The Importance of Genes

TTH and migraine are two common headaches. Because these two types of pain share some common characteristics (trigeminovascular pathology, stress, pain intensity, nausea), distinguishing TTH and migraine is difficult due to the lack of specific diagnostic tests and biomarkers. The distinguishing features of tension-type headache include prevalence, gender differences, attack duration, pain character and lateralization, pain intensity, dorsal root ganglion, nociceptors in the pericranial muscles, nociceptors in the occipital region skin, and nociceptors in the facial skin [13]. The range of effects of the Wingless-related integration site (WNT) signaling pathway included in this study is wide [8]. Genetic markers may be predictive of the causes of pain. In particular, mechanisms such as methylation, which directly affects the functioning of genes, may contribute to TTH pathology. Epigenome-wide studies carried out specifically in migraine were evaluated in patients with chronic migraine and excessive medication use as part of the research to reverse migraine. A relationship was detected between the methylation profile in two genes (*HDAC4* and *MARK3*) and pain [14]. These studies made it possible to reveal the methylation profile in a TTH and provide an appropriate link to its pathology. Thus, whether the genes in our study are methylated or unmethylated may explain the process of revealing their effects on the expression of the genes (*WNT5A* and *FZD3*). It may also enable them to serve as effective therapeutic targets for the treatment of TTH.

### 2.4. Detection of Methylation Status and Design of Primers

Methbank database was used to determine the methylation status of selected genes (Methbank 3.4, Release 4.0, 2022-09) (https://ngdc.cncb.ac.cn/methbank/cpgisland (accessed on 20 November 2023)). The sequences of the investigated genes were determined according to their chromosomal location. In the next step, CpG islands were evaluated according to their average methylation status (>90%) and their genomic locations were accessed according to their high methylation rate. The gene in the map was selected from the action data using the J Browse menu and this sequence was used to predict CpG islands. The selection of CpG island for the *FZD3* gene (Gene ID:7976, Amplicon Length: 235 bp) was based on exon methylations because they affect alternative splicing and the potential for these effects to be reflected in the protein [15]. At the same time, this region has a regulator feature as a silencer (chr8 chr8:28351729-28421152 (+strand) (https://www.ncbi.nlm.nih.gov/nucleotide/NG_179563.1?report=genbank&log$=nuclalign&blast_rank=1&RID=29CTNNU0016 (accessed on 20 November 2023)). The region selected for another gene, *WNT5A* (Gene ID:7474, Amplicon Length: 178 bp), contains promoter methylation (chr3 chr3:55465715-55490539 (−strand)) (https://www.ncbi.nlm.nih.gov/nucleotide/U39837.1?report=genbank&log$=nuclalign&blast_rank=3&RID=29DZU258013&from=1806&to=2177 (accessed on 20 November 2023)). Promoter region methylation status of genes may be related to activation or inactivation of genes. To determine the methylation status using Real-Time PCR for methylation-specific PCR, the primer design was carried out using the online MethPrimer program (http://urogene.org/cgi-bin/methprimer/methprimer.cgi (accessed on 20 November 2023)) and adhering to the general methylation primer design rules for methylation and unmethylation [16].

### 2.5. Study Design

This study evaluated *WNT5A* and its receptor *FZD3* for methylation and gene expression profile in a TTH cohort. The blood samples were taken via peripheral venous access 24 hours after the last pain. A 3 mL whole blood sample was taken from the cubital vein into EDTA tubes from all participants who were followed for at least 6 months and stored at −20 °C. Once the samples were completed, they were studied simultaneously.

### 2.6. DNA Extraction and Methylation Profiling

DNA extraction from whole blood (3 mL) was performed using the PureLink™ Genomic DNA Mini Kit (Invitrogen™, K182000, Waltham, MA 02451, USA). DNA purity (1.78) was determined using nanodrop (Thermo Fisher Scientific, Waltham, MA 02451, USA). The cytosine–thymine conversion was performed with sodium bisulfite and purified with EZ-DNA Methylation-Gold™ Kit (Zymo Research, D5005, Irvine, CA, USA). DNA recovery after bisulfite conversion was detected with a spectrophotometer (Thermo Fisher Scientific, MultiSkan GO, Waltham, MA 02451, USA) and analyzed with the Human MethylLigth PCR Kit according to the commercial protocol (EpiTect, 59496, Qiagen, Redwood City, CA 94063, USA). Methylation-specific quantitative PCR analyses were performed with the TaqMan primer probes. Specific primer sequences were designed for methylated and unmethylated regions (Table 1) for real-time polymerase chain reaction (Real-Time PCR).

Reactions were also analyzed for unmethylated sequences to ensure methylation status. PCR reactions were prepared in 25 μL. The reaction contained 12.5 μL of 2x EpiTect MethyLight Master Mix (Qiagen, Redwood City, CA 94063, USA), 2.5 μL of left and right primer (10–100 pmol/μL in 0.3–3 μM final concentration), 9 μL ddH_2_O, and 1 μL (20 ng) bisulfite-converted template DNA. Products were incubated in a thermal cycler (Rotor-Gene Q, Qiagen, Redwood City, CA 94063, USA) in two steps according to the following temperature profile: 95 °C for 5 min for 1 cycle, 95 °C for 15 s, and 60 °C for 60 s for 40 cycles. Reactions included a no template control and bisulfite-converted methylated-unmethylated positive control DNA. The β-actin (*ACTB*) gene was used for methylation normalization [18]. The β-actin sequence contained no CpG sites. Thus, the gene was unmethylated and always converted to uracil after bisulfite conversion. In calculating the data, the target cycle threshold values (Ct) of each sample were normalized to the ACTB (Ct) (ΔCt = Ct (Target) − Ct(ACTB)) as the reference gene. Relative quantification (RQ) of all samples was converted to standard methylated DNA concentration (0.5%) (RQsample = 2^−ΔΔCt^, ΔΔCt = ΔCt (sample) − ΔCt(calibrator)) [19]. Each analysis was performed in triplicate. Average ΔCt data were used to determine the degree of methylation.

### 2.7. RNA Extraction and Gene Expression Profiling

Analysis of the expression changes of the two genes was performed by taking a 3 mL peripheral whole blood sample from each individual. Total RNA was obtained by modifying the acid guanidinium thiocyanate-phenol-chloroform (AGPC) RNA extraction method [13]. RNA quantification was determined with a spectrophotometer (MultiSkan GO, Thermo Fisher Scientific, Waltham, MA 02451, USA) at 260 nm and 280 nm values (100 ng/µL). RNA purity (2.01) was determined using nanodrop (Thermo Fisher Scientific, USA) (median RIN = 7.3 for control and 7.6 for patient samples). Complementary DNA (cDNA) extraction from total RNA was performed using a reverse transcriptase kit (RT) according to the manufacturer’s recommendations (Quantitect^®^, Reverse Transcription Kit, Qiagene, 205313, Redwood City, CA 94063, USA). The prepared 10 μL cDNA mixture contained 5 ng total RNA, 5x HiSpec Buffer, 10x Nucleic Mix, Reverse Transcriptase Mix, and ddH_2_O water. Reactions were incubated in an automatic thermal cycler (Veriti, Applied Biosystem, Foster City, CA, USA) under the following conditions: 42 °C for 60 min, 95 °C for 5 min, and 85 °C for 5 min. At the end of the period, they were immediately placed on ice and stored at −20 °C until further analysis. Quantitative comparative Real-Time PCR analyses (qRT-PCR) were performed on the Rotor-Gene Q (Qiagen Strasse 1, D-40724 Hilden, Germany) with SYBR Green technology. Gene expressions were normalized to the *ACTB* gene (QT00095431, Qiagen) and a standard RNA control (Thermo Fisher Scientific; AM7155, Waltham, MA 02451, USA). The 20 μL reaction mixture contained 5 μL of RT-PCR product, 2X SYBRGreen PCR Master Mix (Qiagen, Redwood City, CA 94063, USA), *FZD3* (QT00009114, Qiagen, Redwood City, CA 94063, USA), and *WNT5A* (QT00025109, Qiagen, Redwood City, CA 94063, USA) specific primer. Reactions were incubated in a thermal cycler under the conditions given below: two-step PCR was performed with 1 cycle at 95 °C for 15 min, 30 cycles at 94 °C for 5 s, and 55 °C for 60 s (mean Cq value (Cqmean) = 28.5 ± 5.3). All reactions were run in triplicate and calculated according to 2^−ΔΔCt^ [14].

### 2.8. Statistical Analysis

Normality analyses of the data were tested with Shapiro–Wilk tests. The Mann–Whitney U test was used to compare variables that did not comply with normal distribution between groups, and the chi-square test was used to compare categorical variables. Analyses were performed with the SPSS (Release 22.0, SPSS Inc., Chicago, IL, USA) package program and were considered statistically significant when the *p* value was ≤0.05. Receiver operating characteristic (ROC) curve analyses were performed to analyze the expression and methylation status of the genes and the AUC was evaluated.

## 3. Results

### 3.1. Clinical Characteristics

Demographic and clinical characteristics (in serum samples) of the Case–Control group are given in Table 2. Age (*p* = 0.002) and BMI (*p* = 0.034) were statistically significant in comparisons between groups according to the Mann–Whitney U test. Other comorbidities were not significant (*p* > 0.05). Differences in methylation and gene expression for groups were analyzed using one-way ANOVA. The results were statistically significant (*p* < 0.01).

### 3.2. Methylation and Gene Expression Status

Table 3 provides descriptive statistics for methylation and gene expression, and the homogeneity of variances was analyzed using an independent samples *t*-test.

The difference in expression and methylation status of genes was significant between groups (*p* < 0.01). The *FZD3* and *WNT5A* genes were upregulated in TTH patients. *FZD3* showed a hypomethylation profile, while *WNT5A* showed a hypermethylation profile in patients. The results of the correlation analyses were as follows: age–*FZD3* expression (Pearson correlation = −0.209, *p* < 0.001), gender–*FZD3* expression (Pearson correlation = −0.160, *p* = 0.012), gender–*FZD3* methylation (Pearson correlation = −0.228, *p* < 0.001), AST–*FZD3* methylation (Pearson correlation = −0.1419, *p* = 0.027), B12–*FZD3* methylation (Pearson correlation = −0.166, *p* = 0.009), *WNT5A* expression–*WNT5A* methylation (Pearson correlation = 0.425, *p* < 0.001), *WNT5A* expression–*FZD3* expression (Pearson correlation = 0.246, *p* < 0.001), *WNT5A* expression–*FZD3* methylation (Pearson correlation = 0.404, *p* < 0.001), *WNT5A* methylation–*FZD3* expression (Pearson correlation = 0.329, *p* < 0.001), and *FZD3* expression–*FZD3* methylation (Pearson correlation = 0.442, *p* < 0.001). When the co-efficiencies between the groups were evaluated, it was detected that the disease could be predicted with a higher sensitivity depending on the methylation and expression of the genes. When the co-efficiencies between the groups were evaluated, it was observed that, depending on the methylation and expression of the genes, the methylation of *WNT5A* could predict the disease with a higher sensitivity than the expression (coefficient SE_WNT5Aexpression_ = 0.002, *p* = 0.743; coefficient SE_WNT5Amethylation_ = 0.001, *p* = 0.008), and *FZD3* could predict the disease with equal sensitivity for both expression and methylation (coefficient SE*_FZD3_*_expression_ = 0.004, *p* < 0.001; coefficient SE*_FZD3_*_methylation_ = 0.001, *p* < 0.001).

### 3.3. ROC Analysis for Diagnostic Power

**Figure 1:**
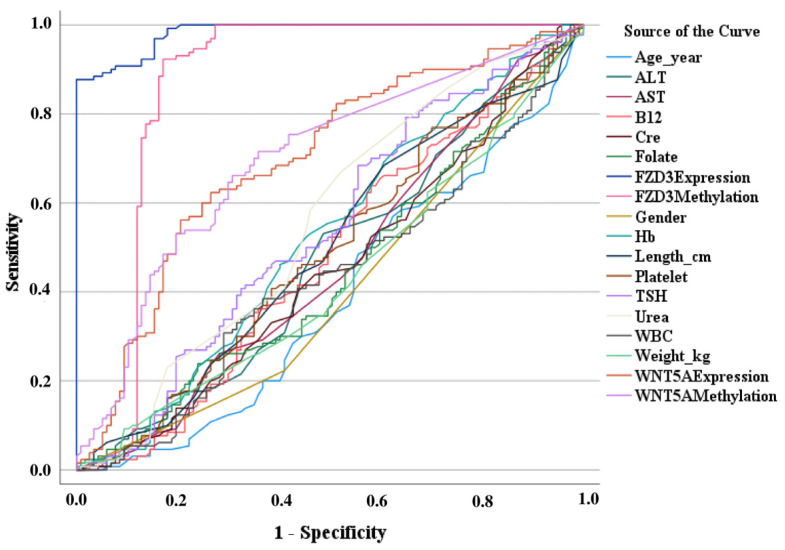
Diagnostic power of parameters with ROC analysis.

The ROC analyses showed the diagnostic power of comorbidities and differences in gene expression and methylation changes between groups (Figure 1) and AUC values (Table 4).

The AUC data showed that age and gender did not have diagnostic power, *WNT5A* expression and methylation were satisfactory in diagnosing patients, and *FZD3* expression was excellent and methylation was very good.

## 4. Discussion

DNA methylations host striking epigenetic mechanisms in the genome. Memories programmed from the beginning of fetal life may change during the zygotic stage, and some memories may remain while others may be erased. An important point is that genes may become silent at the cellular and textural levels or require interpretation. Errors in the regulation of this clock can disrupt the methylation program. The basis of human diseases is generally explained through gene expressions and polymorphisms. Since the DNA code is almost completely stable, it is undeniable that a definitive answer can be obtained when there is a change. However, epigenetic elements, such as miRNAs and methylation, which have gene regulation potential, are important in the phenotypic reflection of genes. In migraine, where a gene cannot be held responsible as in neurological diseases, some studies have included genetic sources related to many symptoms, from the form of patients with and without aura to the number of migraine attacks. The effects of this condition can only be monitored clinically and in multifactorial diseases. When considered as headaches in general, the situation is more complicated. Especially today, the increase in the number of triggers makes it difficult to attribute the causes of the disease to a genetic basis. Among the causes of headache, the tension type is the group in which the influence of the environment is felt most clearly. Therefore, in our research, we analyzed the methylation profile of WNT5A and its receptor FZD3, one of the systemic epigenetic servers, in response to the environment and the expression changes in related genes and evaluated their contribution to the TTH process together with clinical findings. Revealing expressional differences according to the methylation status of genes may help control a TTH through epigenetic mechanisms. At the same time, they can be used as diagnostic markers in the clinical diagnosis of the disease.

The most common type of headache is a TTH. Causes of a TTH include stress and muscle tone. TTH symptoms are generally felt as constant pain. A TTH resembles migraine attacks because it is characterized by mild to moderate bilateral attacks. It is difficult to attribute the close clinical features and causes of these two diseases to a specific genetic factor. Therefore, epigenetic changes may provide an important resource. When we look at the clinical picture of the disease, the pain starts slowly, is bilateral, and feels like a tightness, and mild to moderate pain can be cured depending on the cause. But when there are genetic connections, it will not allow recovery. Moreover, their transmissibility can change the course and severity of the disease. Familial hemiplegic migraine is evidence of this because it is the determinant of intergenerational probability with its autosomal dominant character. Increased pericranial myofascial pain sensitivity, spinal dorsal horn, sensitized trigeminal nucleus second-order neurons, and inhibition in supraspinal structures are factors in TTH [20]. Within this mechanism, WNT has a high responsibility because it plays a role in embryonic development and the regulation of the nervous system [8]. WNT5A- and FZD3-induced changes in the embryonic period may pave the way for patients to be diagnosed with TTH at a young age. Treatments are not definitive and provide temporary relief. The basis of acute therapy is aspirin and non-steroidal inflammatory drugs (NSAIDs). Past randomized studies show that aspirin and acetaminophen are effective in acute therapy [15]. Tricyclic antidepressants are another treatment agent. In addition to pharmacological therapies, patient education, and lifestyle modification are among the supportive treatments [16]. Comorbidities in TTH (neck pain and low back pain) should not be overlooked [18]. Otherwise, explaining epigenetic changes in diseases that are affected by environmental factors, such as drugs, nutrients, and stress, may not be possible. The pathophysiological mechanisms involved in TTH are complex. However, family studies indicate that the risk is significant in first-degree relatives [19]. Concordance and discordance studies provide interesting answers to the question of whether genetic or environmental influences are effective. Data obtained from monozygotes and dizygotic showed that the association of TTH with a young age, female gender, and high body mass index was due to sharing the same environmental conditions [21,22,23]. Sudden and delayed headache initiates the nitric oxide (NO)-mediated biphasic response that is typical in TTH. A study conducted in the middle age group shows that the T allele in the rs3782218 polymorphism in the NOS1 gene has biomarker potential in TTH [24]. The possible responsible genes are the serotonin transporter gene-linked polymorphic region (*5-HTTLPR*) and the Val158Met polymorphism in the *COMT* gene, which are held responsible for the chronic onset of TTH [25]. The APOE-ε4 gene is recommended as protective against TTH [26]. However, there is no definitive candidate that is responsible and could be a treatment target. Genome-wide association studies (GWAS) report that genes that are candidates for migraine (*LPR1* and *FHL5*) may also be candidates for TTH (a genome-wide association study found genetic associations with broadly defined headache in the UK Biobank (N = 223,773)) because the majority (90%) of migraine patients suffer from TTH [27].

DNA methylation was investigated in a group that transformed from episodic headache to chronic headache. A strong association was found between headache and CpG methylation in the *SHD5* and *NPTX2* genes, which are responsible for the regulation of synaptic plasticity. There is a need for methylation-based studies in this area. If methylation changes the functioning of the genes in which they are located, investigating their expression could provide important data regarding their connection with the disease.

The effects and effect size of CpG islands in the genome vary depending on their location. Abnormally methylated CpG islands located in the 5′ promoter region of genes have been associated with the transcriptional inactivation of various genes in cancer. By detecting DNA methylation profiles, they can be potential biomarkers in diseases and serve as a potential target for the development of antimethylation therapeutic strategies. WNT5A is important in embryonic development and nervous system regulation. It is a potential therapeutic target within the broad spectrum of pain (migraine, neuropathic pain, endometriosis pain, sciatic nerve pain) [8].

When the effect of WNT5A is evaluated under the heading of pain, it is seen that it has a managerial role in the dendritic spine [28] and astrogliosis [29] since there is a diversity of pain classes. When the pathophysiology of TTH is drawn, the picture that emerges suggests that myofascial trigger points and excessive contractions may remain latent after ischemia [30]. In addition, myodural bridges may form as a result of the contraction of the upper neck muscles and the pulling of the dura material [31]. Studies on the relationship between the environment and genes show the importance of genetic factors in TTH [23]. Additionally, the closest relationship is described for the *NOS1*, *NOS2*, and *NOS3* genes. Some polymorphisms in these genes are thought to be associated with a susceptibility to TTH [24]. Although *WNT5A* has been most recently described in pain processes, its expression–methylation relationship has not been defined in TTH. Evidence during osteoblastic differentiation shows ovarian expression of *WNT5A*. In the absence of the protein, changes in the cell cycle, proliferation, growth, and transcriptional regulation of genes are observed [32]. Our study evaluating *WNT5A* expression also shows that it is overexpressed in TTH patients. Hypermethylation of the gene in TTH patients, in whom we examined promoter methylation, indicates that the relevant factors may not provide expressional control for transcriptional silencing. This result is important in selecting treatment targets because access to a protected promoter can be difficult. WNT5A enhances Wnt/β-catenin signaling [33]. In particular, it highlights the role of WNT5A in several pathological pain disorders. The abnormal activation of WNT signaling increases neuroinflammation, reduces intraepidermal nerve fiber density by affecting synaptic plasticity, and contributes to chronic pain (Wnt signaling: a prospective therapeutic target for chronic pain). Therefore, the overexpression of *WNT5A* due to hypermethylation may contribute to TTH pathology by triggering Wnt/β-catenin signaling. In our analysis for *FZD3*, which involved the WNT5A receptor, upregulation was detected. Upregulation of the gene is encountered in cancer [34]. However, its importance in the pain process has not been mentioned. WNT signaling is responsible for the establishment and functional regulation of neuronal circuits. As receptors for WNTs, FZDs are responsible for neuronal processes (neurogenesis, axon guidance, dendritogenesis, synapse formation, and synaptic plasticity) [34]. The data obtained in this study show that hypomethylation modulates the *FZD3* function in the patient. This may result in changes in WNT signaling in neurons. It can be considered that exon methylations are not as effective as promoter methylations. Researchers are still in doubt about its direct effects, but there are predictions that the mechanism we encounter here is splicing. Another issue is the silencers found in the exons. DNA methylation may affect splicing, but not for all exons because the distribution of CpG islands may not correspond to all exons in a gene. A study using a genome-wide approach showed that more than 20% of alternative exomes were affected by a lack of DNA methylation [35]. These exons served as splicing enhancers or silencers. Our *FZD3* methylation region is in an exon that has regulatory properties as a silencer. It is possible that it lost its silence through hypomethylation. This result, which causes excessive activation of the receptor, may cause a burden on the pain process through excessive stimulation of WNT5A activation and signaling.

## 5. Conclusions

TTH is common in society and environmental factors are more effective than for other types of pain. No difference was observed in serum data, allowing the patients to be examined solely on a genetic basis, excluding B12, folate, TSH, and other important markers. Because the direct effect of drugs on methylation and gene expression is low, two important genes of the Wnt pathway, *WNT5A* and *FZD3*, and their methylation status can be significant in TTH patients. In addition, *FZD3* expression and methylation, which have a high diagnostic value, have biomarker potential to identify these patients. Further analysis may help regulate gene activities by targeting methylation profiles. Investigating the diagnostic value in larger groups may be helpful for early medication. A limitation of this study is that the groups were not matched for gender.

## Figures and Tables

**Table 1:** Primer and probe sequence for *FZD3* and *WNT5A* gene methylation profile detection.

Primer Probe Name ^1^	*FZD3* Sequence
Left meth primer	GTTATAATAAAAGTTTTGGGCGTC
Right meth Primer	AAATCTAAACCAAATAAAAACGAA
Probe meth	FAM-GCGGGCTGCGGAGCGGGGGCCCGGGCCCGC-MGB
Left unmeth primer	TTATAATAAAATTTTGGTGTTGG
Right unmeth primer	CAAAATCTAAACCAAAATAAAAACAAA
Probe unmeth	VIC-GCGGGTTGCGGAGCGGGGGTTCGGGTTCGC-MGB
	***WNT5A* sequence**
Left M primer	TTGAAGAACGTTTTTAAGGAAATTC
Rigth M Primer	AAAAAAAACAAAAAATAAAAAACGA
Probe meth	FAM-GCTGCATTTCCCAGCTGGGCACTCTCGCGCGC-MGB
Left unmeth primer	TTGAAGAATGTTTTTAAGGAAATTTG
Right unmeth primer	AAAAAAAACAAAAAATAAAAAACAA
Probe unmeth	VIC-GTTGTATTTTTTAGTTGGGTATTTTCGCGCGT-MGB
	***ACTB* sequence** [17]
Left M primer	TGATGGAGGAGGTTTAGTAAGTTTT
Rigth M Primer	CACCACCCAACACACAATAACAA
Probe meth	FAM-TGGATTGTGAATTTGTG-MGB

^1^ Meth; methylated, unmeth; unmethylated. ***ACTB* sequence referred to** [17].

**Table 2:** Clinical characteristics of study groups.

	Control (N, %)	TTH (N, %)
Number of groups	117	130
Age (years)		
17–30	37 (31.63)	51 (39.23)
31–45	30 (25.64)	41 (31.54)
46–60	24 (20.51)	30 (23.08)
61–75	24 (20.51)	8 (6.15)
76–82	2 (1.71)	0
Gender (N, %)		
Female	69 (58.97)	101 (77.69)
Male	48 (41.03)	29 (22.31)
	**Median (25–75%)**	**Median (25–75%)**
Length (cm)	165 (163–172)	165 (163–169)
Weight (g)	72 (65–78)	72 (65–78)
BMI (kg/m^2^)	25.59 (24.46–27.31)	24.8 (23.03–26.57)
Hemoglobin (g/L)	13.85 (12.7–14.9)	13.6 (12.4–14.9)
WBC (×10^3^ per μL)	7.64 (6.6–9.33)	7.45 (6.08–8.86)
Platelet (×10^3^ per μL)	274 (238–342)	273.5 (243–327)
Urea (mmol/L)	21 (19.24)	23 (20–25)
Creatinine (mg per dL)	0.658 (0.59–0.8)	0.66 (0.57–0.75)
AST (IU per L)	22 (18–26)	20 (18–25)
ALT (IU per L)	18 (14–26)	19 (14–24)
B12 (ng/L)	235 (175–365)	230 (180–324)
Folate (μg/L)	7.7 (5.86–9.43)	6.74 (5.66–9.32)
TSH (mU/L)	1.56 (1.03–2.12)	1.59 (1.21–2.14)
		**Median ± SD**
Number of pain occurrences (number in one month)	None	10.74 ± 8.49
Severity of attacks	None	3.71 ± 1.73
Use of analgesic (number in one month)	None	10.35 ± 12.34
		**N (%)**
Location of pain	None	
right side		8 (6.15)
parietal		12 (9.23)
widespread		42 (32.31)
bitemporal		13 (10.00)
frontal		18 (13.85)
occipital		32 (24.61)
left side		5 (3.85)
Alcohol	None	
Yes		1 (0.77)
No		129 (99.23)
Cigarette smoke	None	
Yes		14 (10.78)
No		116 (89.23)
Asthma	None	
Yes		5 (3.80)
No		125 (96.20)
Chronic lung disease	None	
Yes		6 (4.60)
No		124 (95.40)
Diabetes Mellitus	None	
Yes		2 (1.50)
No		128 (98.50)
Hypertension	None	
Yes		11 (8.50)
No		119 (91.50)
Hyperthyroidism	None	
Yes		3 (2.30)
No		127 (97.70)
Hypothyroidism	None	None
Hepatitis B	None	
Yes		4 (3.10)
No		126 (96.90)
Hepatitis C	None	None

TTH, tension-type headache; SD, standard deviation; N, number of individuals.

**Table 3:** Differences of groups for gene methylation and expression.

Gene	Status	Groups	N	Mean ± SE	SD	95% CI for Mean	*p*-Value
*FZD3*	Expression	Control	117	6.370 ± 0.162	1.754	6.049–6.692	<0.01 *
		Patient	130	14.145 ± 0.296	3.379	13.559–14.732	
	Methylation	Control	117	11.096 ± 0.508	5.494	−12.102–−10.090	<0.01 *
		Patient	130	−5.158 ± 0.228	2.597	−5.609–−4.708	
*WNT5A*	Expression	Control	117	−5.100 ± 1.220	13.202	−8.413–−3.578	<0.01 *
		Patient	130	3.014 ± 1.078	12.293	0.881–5.148	
	Methylation	Control	117	18.068 ± 2.114	22.862	13.882–22.255	<0.01 *
		Patient	130	35.043 ± 1.961	22.356	31.163–38.922	

* *p* < 0.01 is statistically significant. N, the number of individuals; SE, standard error; SD, standard deviation; CI, confidence interval. Bold expressions indicate statistical significance.

**Table 4:** AUC values of comorbidities.

Comorbidities	Area	SE	*p*-Value	95% CI
Age (year)	0.388	0.036	0.002 *	0.317–0.458
Gender	0.406	0.036	0.011 *	0.335–0.478
Length (cm)	0.494	0.037	0.875	0.421–0.567
Weight (kg)	0.429	0.036	0.052	0.357–0.500
Hb (g/L)	0.526	0.037	0.489	0.453–0.599
WBC (×10^3^ per μL)	0.438	0.037	0.092	0.366–0.510
Platelet (×10^3^ per μL)	0.489	0.037	0.764	0.416–0.562
Urea (mmol/L)	0.557	0.037	0.122	0.484–0.630
Creatinine (mg per dL)	0.452	0.037	0.189	0.379–0.524
AST (IU per L)	0.463	0.037	0.314	0.390–0.536
ALT (IU per L)	0.473	0.037	0.456	0.400–0.545
B12 (ng/L)	0.478	0.037	0.544	0.405–0.551
Folate (μg/L)	0.446	0.037	0.140	0.374–0.518
TSH (mU/L)	0.540	0.037	0.283	0.467–0.612
*WNT5A* expression	0.697	0.034	0.0009 *	0.630–0.763
*WNT5A* methylation	0.697	0.034	0.0001 *	0.631–0.763
*FZD3* expression	0.983	0.005	0.0002 *	0.973–0.994
*FZD3* methylation	0.866	0.029	0.0003 *	0.808–0.923

* *p* < 0.05 is significant. SE, standard error; CI, confidence interval. Bold expressions indicate statistical significance.

## Data Availability

The datasets generated during and/or analyzed during the current study are available from the corresponding author upon reasonable request.

## Abbreviations

5-HTTLPR	5HTt Gene-Linked Polymorphic Region
ACTB	Actin Beta
AUC	Area Under the Curve
APOE-ε4	Apolipoprotein E4
CALCA	Calcitonin Related Polypeptide Alpha
COMT	Catechol-O-methyltransferase
CT	Computational Tomography
FHL5	Four And A Half LIM Domains 5
FZD3	Frizzled Class Receptor 3
GWAS	Genome-Wide Association Study
